# Mechanisms of spinal glial activation in chemotherapy‐induced peripheral neuropathy: Focus on microglia and astrocytes

**DOI:** 10.1002/ibra.70007

**Published:** 2025-12-02

**Authors:** Long Gu, Song Cao, Yonghuai Feng

**Affiliations:** ^1^ Department of Hematology Affiliated Hospital of Zunyi Medical University Zunyi China; ^2^ Department of Pain Medicine The Tenth Affiliated Hospital, Southern Medical University (Dongguan People's Hospital) Dongguan China; ^3^ Dongguan Key Laboratory of Anesthesia and Organ Protection Dongguan China; ^4^ Department of Hematology The Tenth Affiliated Hospital, Southern Medical University (Dongguan People's Hospital) Dongguan China

**Keywords:** astrocytes, chemotherapy‐induced peripheral neuropathy, microglia, neuropathic pain, peripheral nerve injury

## Abstract

Chemotherapy‐induced peripheral neuropathy (CIPN) is a common complication in patients with malignant tumors during chemotherapy. The pathological mechanisms of CIPN remain unclear, and effective preventive and therapeutic strategies are still lacking, posing a major challenge in clinical practice. Aberrant activation of spinal glial cells, particularly microglia and astrocytes, is a key pathological hallmark of CIPN. Evidence from multiple animal models supports a causal link between glial activation and CIPN, suggesting that glial cells may serve as potential therapeutic targets. However, owing to the diversity of chemotherapy agents, the mechanisms of glial activation in CIPN differ and remain insufficiently characterized. This review takes spinal glial activation induced by peripheral nerve injury as its starting point, with a specific focus on microglia and astrocytes. It provides a systematic overview of their roles and mechanisms in CIPN caused by commonly used chemotherapeutic agents. The aim is to deepen understanding of CIPN pathogenesis and provide a foundation for developing targeted therapies.

## Graphical abstract

Chemotherapy‐induced peripheral neuropathy (CIPN) remains a major clinical challenge, with central sensitization considered an important pathological mechanism in its occurrence and persistence. This review focuses on spinal astrocytes and microglia, systematically summarizing the pathological mechanisms of spinal glial cells involved in CIPN caused by commonly used chemotherapeutic agents (paclitaxel, vincristine, oxaliplatin, cisplatin, and bortezomib), aiming to enhance understanding of CIPN pathogenesis and provide insights for targeted therapeutic strategies.

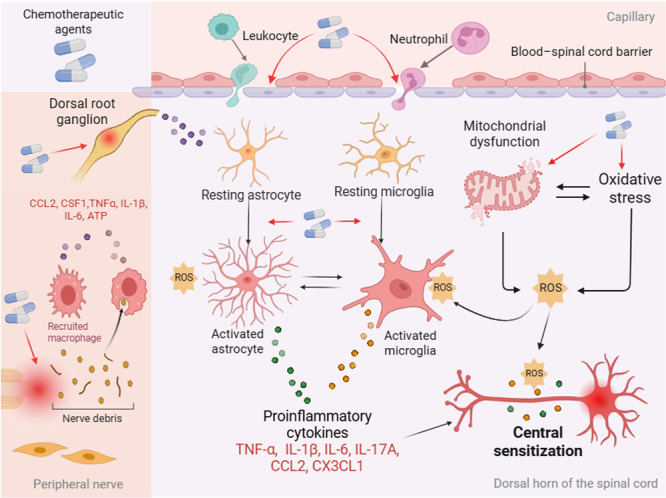

## 1 INTRODUCTION

Malignant tumors are among the most common diseases threatening human health worldwide, and chemotherapy, as a major treatment modality, is widely applied in clinical practice.^1^ Chemotherapy‐induced peripheral neuropathy (CIPN) is a frequent adverse effect of chemotherapeutic agents that profoundly affects patients' daily lifeand treatment adherence.^2^ Some patients are compelled to adjust chemotherapy regimens or even discontinue treatment due to CIPN, thereby significantly reducing the survival benefits of cancer therapy.^3^ Unfortunately, the pathophysiological mechanisms of CIPN remain poorly understood, and effective interventions are lacking.^4^ Although duloxetine is recommended for the relief of painful CIPN, its efficacy is limited and it may cause adverse effects such as dizziness, insomnia, and nausea.^5^ Therefore, a deeper understanding of the mechanisms underlying CIPN and the development of targeted therapeutic strategies are of critical importance in clinical practice. Notably, aberrant activation of spinal glial cells has emerged as a prominent area of CIPN research.

Growing evidence indicates that glial cells play a critical role in the initiation and persistence of neuropathic pain.^6^, ^7^ Under pathological conditions, glial activation promotes the release of inflammatory mediators and neuroactive substances, markedly amplifying neuronal pain signals and driving the onset, persistence, and exacerbation of pain symptoms.^8^, ^9^ Glial cells were once regarded merely as structural and functional supporters of neurons. However, accumulating research has demonstrated that they are essential for regulating neural signal transmission, repairing neuronal injury, and maintaining ion homeostasis.^10^ Glial inflammatory responses are strongly implicated in a range of neurological disorders,^11^, ^12^, ^13^ including neurodegenerative diseases, cerebral ischemia, and multiple sclerosis.^14^, ^15^, ^16^ Similarly, in the development and progression of CIPN, activation of astrocytes and microglia is considered highly significant. These glial cells not only mediate neuroinflammatory responses but also exacerbate neuronal injury and pain perception by modulating neuronal function and pain transmission pathways.^17^, ^18^, ^19^, ^20^ Therefore, to better elucidate the molecular mechanisms by which glial cells mediate CIPN, as well as their interactions and regulatory patterns, we provide a systematic overview of recent advances in this field. This review summarizes the features and underlying mechanisms of astrocytic and microglial activation in CIPN induced by different chemotherapeutic agents.

## 2 CLINICAL MANIFESTATIONS AND MECHANISMS OF CIPN

**Figure 1:**
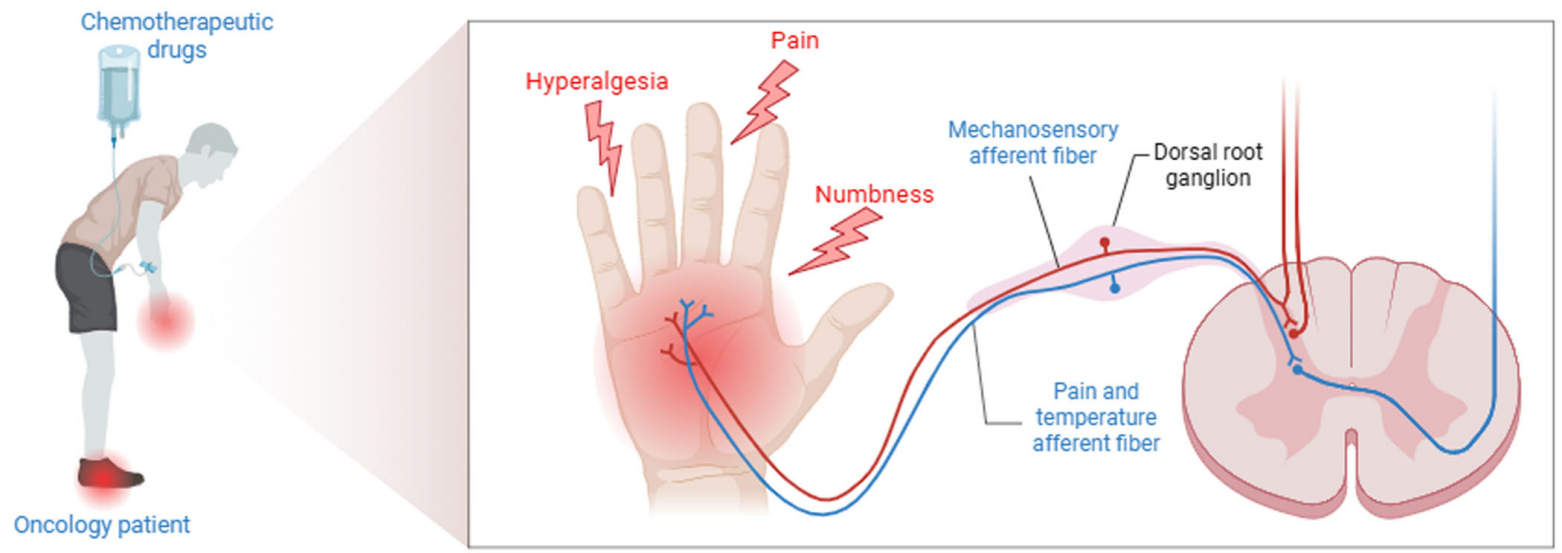
Distal limb sensory abnormalities and afferent pathways in CIPN patients. Cancer patients receiving chemotherapy (left) often develop distal sensory abnormalities (middle), including dysfunction of mechanical sensation and pain/temperature perception. Blue lines indicate afferent fibers transmitting mechanical sensation, while red lines represent afferent fibers transmitting pain/temperature signals. These fibers convey peripheral inputs through the dorsal root ganglion to the dorsal horn of the spinal cord. CIPN, chemotherapy‐induced peripheral neuropathy.

With the rising incidence of malignant tumors and the widespread use of chemotherapy, the prevalence of CIPN has increased significantly.^1^, ^21^ Multiple first‐line chemotherapeutic agents for both solid and hematologic malignancies can induce CIPN.^22^ The main clinical features of CIPN are symmetrical sensory abnormalities in the distal extremities, such as pain, numbness, and tingling (Figure 1); in severe cases, motor nerve involvement may occur, leading to muscle weakness and balance impairment. The disease course is typically progressive, persistent, and irreversible.^23^ Symptoms are categorized as acute or chronic. Approximately 90% of patients experience at least one acute neuropathy symptom during the first chemotherapy cycle, whereas the incidence of chronic CIPN (13%–70%) varies according to the type of chemotherapeutic agent and cumulative dose.^24^ Neuropathic pain is the predominant symptom of CIPN, with an incidence of up to 80%.^25^ This pain correlates with the cumulative dose of chemotherapeutic agents and progressively worsens during treatment, substantially impairing patients' quality oflife. In some patients, pain persists even after chemotherapy completion, eventually progressing to chronic neuropathy.^2^, ^26^

Recent studies have shown that the development of chronic pain, including CIPN, is largely associated with central mechanisms.^27^, ^28^ The spinal dorsal horn is a key center for processing sensory information.^29^ Signals generated by peripheral nerve injury (PNI) are transmitted through the dorsal root ganglia to the spinal dorsal horn, where they activate glial cells and trigger the release of multiple pro‐inflammatory mediators that contribute to nociceptive transmission and central sensitization in the spinal cord.^7^, ^30^ Central sensitization refers to the heightened responsiveness of nociceptive neurons to normal afferent stimuli and represents an important mechanism underlying hyperalgesia and allodynia.^31^, ^32^ In addition, paclitaxel and platinum‐based chemotherapeutic agents can disrupt the blood–nerve barrier, leading to infiltration of peripheral inflammatory mediators and immune cells into the spinal dorsal horn.^33^, ^34^ Neuroinflammation is considered one of the core mechanisms of CIPN. On the one hand, chemotherapeutic agents can directly cause immune dysregulation and trigger neuroinflammation responses.^4^, ^35^, ^36^ On the other hand, microglia and astrocytes in the spinal dorsal horn become activated after PNI and subsequently release various pro‐inflammatory cytokines. These mediators further enhance the excitability of central nociceptive pathways, thereby sustaining persistent pain associated with nerve injury.^37^, ^38^, ^39^, ^40^ Notably, glial neuroinflammation induced by different chemotherapeutic agents varies in both its characteristics and underlying mechanisms.^41^, ^42^, ^43^, ^44^

## 3 THE ROLE OF GLIAL CELLS IN PAIN MAINTENANCE

Neuropathic pain is a chronic pain condition caused by damage to or disease of the somatosensory nervous system. It is typically accompanied by sensory disturbances such as numbness and tingling, and responds poorly to conventional analgesic treatments.^45^, ^46^ The onset and persistence of neuropathic pain are linked not only to neuronal injury but also to glial cell activation and the resulting neuroinflammation.^47^ PNI can trigger dynamic activation of central glial cells, which contribute to pain signal transmission and neural remodeling, serving as a key driving force in the chronic progression of neuropathic pain.^48^, ^49^

As resident immune sentinels of the central nervous system, microglia undergo activation that serves both as a hallmark and an early event in the onset and progression of neuropathic pain.^50^ Damaged peripheral sensory neurons upregulate colony‐stimulating factor 1 (CSF1), which binds to microglial surface receptors and induces their proliferation and activation.^51^ Activated microglia release multiple inflammatory mediators, thereby increasing neuronal excitability and recruiting additional microglia to the injury site, forming a neuroinflammatory positive feedback loop that amplifies the initial injury signals.^52^, ^53^, ^54^ These inflammatory mediators also open volume‐regulated anion channels (VRAC), leading to ATP release that activates spinal purinergic signaling pathways and exacerbates central pain sensitization. Notably, the key VRAC subunit SWELL1 is upregulated in response to PNI.^55^ In addition, the microglia‐specific transient receptor potential cation channel subfamily V member 4 (TRPV4) mediates the transmission of PNI signals to the central nervous system, promoting central sensitization and chronic pain.^56^ Following PNI, microglia have also been reported to selectively degrade perineuronal nets (PNNs) surrounding lamina I neurons in the spinal cord, leading to disinhibition of excitatory neurons projecting to the medullary parasympathetic nuclei and thereby amplifying nociceptive output.^57^ The persistent inflammatory environment further induces astrocytes to shift from homeostatic supporters to active participants in pain regulation, where they play an important role in long‐term maintenance.^58^ After PNI, astrocytes secrete chemokines such as C–C motif chemokine ligand (CCL2) and C‐X‐C motif chemokine ligand 1 (CXCL1) to promote microglial recruitment and enhance neuronal excitability, while also releasing pro‐inflammatory cytokines, including interleukin (IL)‐1β and IL‐6, to further increase synaptic excitatory transmission.^59^, ^60^ Meanwhile, astrocyte‐enriched glutamate transporters (glutamate transporter 1 (GLT1) and glutamate aspartate transporter (GLAST)) are markedly downregulated, leading to glutamate accumulation in the synaptic cleft. This excess glutamate aberrantly activates N‐methyl‐d‐aspartate (NMDA) and alpha‐amino‐3‐hydroxy‐5‐methyl‐4 isoxazoleproprionic acid (AMPA) receptors, resulting in central sensitization through an excitatory‐inhibitory imbalance.^61^ Numerous studies have demonstrated that, in various PNI models, inhibiting microglial activation can effectively prevent hyperalgesia and attenuate nerve injury.^54^, ^55^, ^56^, ^62^ Likewise, blocking specific signaling pathways in astrocytes can also significantly alleviate pain hypersensitivity following nerve injury.^59^, ^63^, ^64^

**Figure 2:**
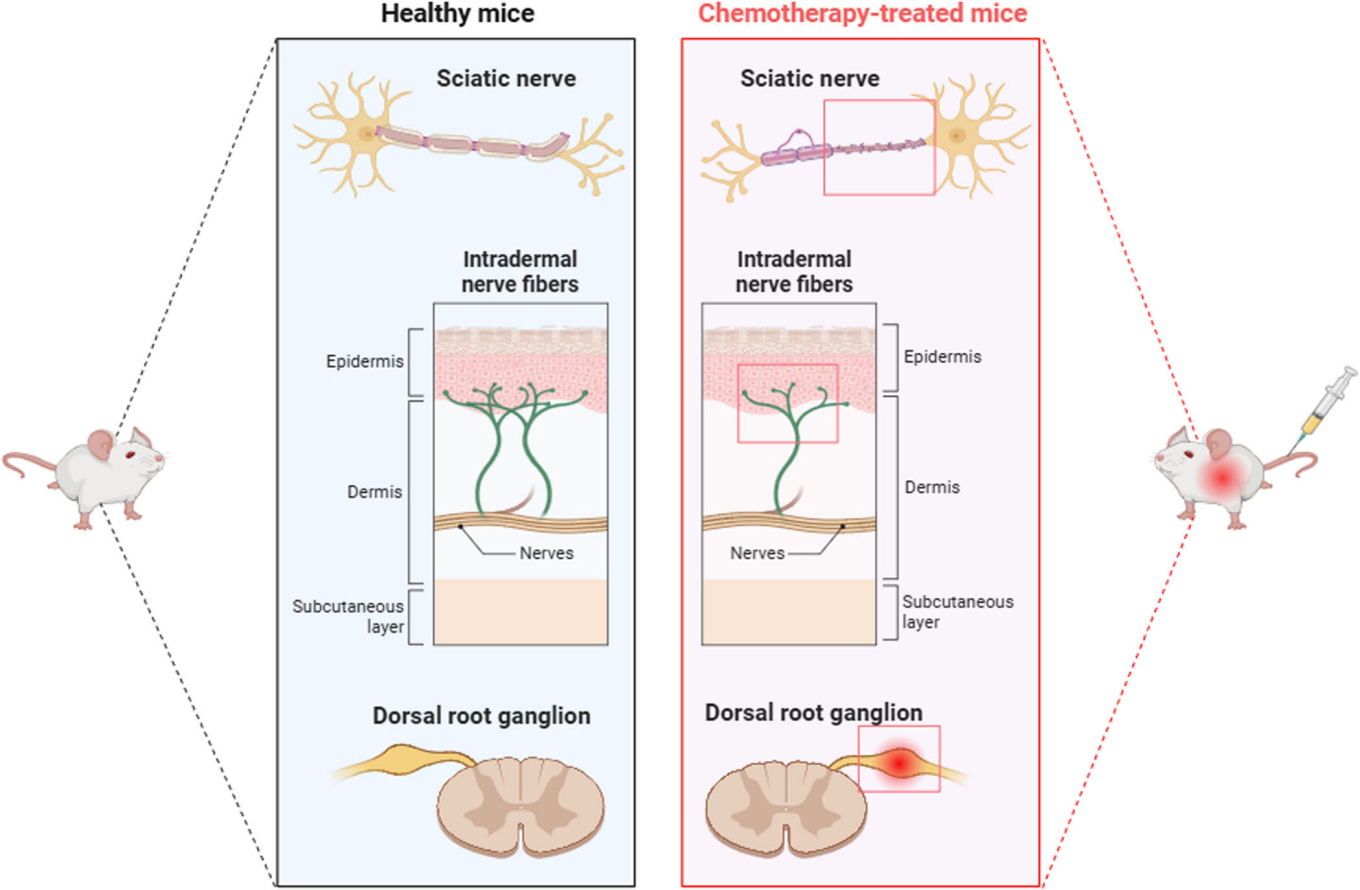
Peripheral nerve damage in preclinical models of chemotherapy‐induced peripheral neuropathy (CIPN). The schematic illustrates structural differences in peripheral nerves between healthy mice (left) and chemotherapy‐treated mice (right). In healthy mice, intraepidermal nerve fibers, the sciatic nerve, and the dorsal root ganglion remain intact. In contrast, chemotherapy‐treated mice show reduced intraepidermal nerve fibers, axonal degeneration and demyelination in the sciatic nerve, and neuronal injury in the dorsal root ganglion. These changes collectively reflect peripheral nerve injury features in preclinical models of CIPN.

In summary, microglia and astrocytes in the central nervous system are pivotal for the initiation and amplification of pain signaling after PNI. Because chemotherapeutic agents can induce PNI (Figure 2), the evidence regarding glial activation and pain maintenance in this context provides a theoretical basis for understanding glial activation in CIPN.

## 4 THE ROLE OF NEUROGLIA IN CIPN AND ITS UNDERLYING MECHANISMS

In CIPN, aberrant activation of microglia and astrocytes has been demonstrated by numerous studies. Whether in CIPN or other forms of PNI, persistent glia‐mediated neuroinflammatory responses are recognized as a key mechanism underlying chronic pain sensitization, although CIPN exhibits distinct mechanistic features. The following sections will discuss glial activation and the key signaling pathways implicated in CIPN induced by several commonly used chemotherapeutic agents, and will conclude with a summary (Table 1).

**Table 1:** Spinal glial cell‐mediated pathological mechanisms and corresponding interventions in chemotherapy‐induced CIPN.

Mechanism	Drug name	Glial type	Therapeutic intervention	Ref.
TLR4/NF‐κB	Paclitaxel	Astrocyte	LPS‐RS, Fecal microbiota transplantation	[65, 66]
		Both	Electroacupuncture treatment	[67, 68]
	Vincristine	Astrocyte	Celastrol, l‐CDL,	[19, 69]
		Microglia	Morin	[70]
	Oxaliplatin	Astrocyte	Diosgenin, PEA‐OXA, Emodin	[71, 72, 73]
		Both	PAM‐2 (a positive allosteric modulator of α7 nicotinic acetylcholine receptors)	[74]
	Cisplatin	Microglia	AIBP	[75]
	Bortezomib	Astrocyte	Berberine	[76]
MAPK	Vincristine	Microglia	Gastrodin	[77]
		Both	Exogenous induction of HO‐1	[78]
	Oxaliplatin	Microglia	Syringaresinol	[79]
	Bortezomib	Astrocyte	Thalidomide and IL‐1ra	[80]
Endocannabinoid system	Paclitaxel	Microglia	Cannabidiol	[81]
		Both	Hyperbaric oxygen	[82]
	Cisplatin	Both	JZL195 (a dual inhibitor of fatty acid amide hydrolase and monoacylglycerol lipase)	[83]
Oxidative stress	Vincristine	Astrocyte	Celastrol, L‐α‐aminoadipate (an astrocyte‐specific inhibitor)	[19, 84]
	Oxaliplatin	Astrocyte	2‐Bromopalmitate, *Hypericum perforatum* L., Resveratrol, NAC, ALA, Vitamin E, Rosmarinic acid	[85, 86, 87, 88, 89]
		Microglia	Magnesium, manganese, and zinc salts	[90]
Adenosine receptor	Oxaliplatin	Astrocyte	A3 adenosine receptor agonist: MRS5698 and IB‐MECA	[91, 92]
		Microglia	Antioxidant‐Conjugated 1,2,4‐Triazolo[4,3‑a]pyrazin‐3‐one Derivatives	[93]
	Cisplatin	Both	Highly selective A3 adenosine receptor agonist: MRS5980	[94]
Imbalance in glutamate homeostasis	Paclitaxel	Astrocyte	Minocycline (a glial activation inhibitor), Valproic acid, Lithium (a GSK3β inhibitor)	[95, 96, 97]
	Cisplatin and Oxaliplatin	Astrocyte	Minocycline	[98]
	Bortezomib	Astrocyte	Minocycline, Carbenoxolone (a gap junction decoupler), ceftriaxone (a glutamate transporter upregulator)	[44]
S1P/S1PR1	Paclitaxe and Bortezomib	Astrocyte	FTY720	[99, 100]
TRPV1	Paclitaxel	Both	Duloxetine, Electroacupuncture treatment	[67, 101]
	Oxaliplatin	Astrocyte	JI017 (an herb mixture composed of *Aconitum carmichaelii*, Angelica gigas, and *Zingiber officinale*), Huachansu (an aqueous extract from toad skin)	[102, 103]
Notch	Paclitaxel	Astrocyte	DAPT (an inhibitor of Notch signaling)	[104]
	Vincristine	Microglia	DAPT	[105]
C5aR1	Paclitaxel	Both	DF3966A (a selective allosteric inhibitor of C5aR1)	[106]
RIP3/MLKL	Paclitaxel	Microglia	Minocycline	[107]
PGE2‐EP2	Paclitaxel	Microglia	Allopregnanolone	[108]
Wnt/β‐Catenin	Vincristine	Both	IWR (inhibitors of Wnt response)	[41]
C/EBP‐β/TGF‐β1	Vincristine	Astrocyte	‐	[109]
CGRP	Cisplatin	Both	ZR8 mAb (a monoclonal antibody targeting CGRP)	[36]
P2Y12/IL‐18	Cisplatin	Microglia	‐	[110]
TREM2/DAP12	Cisplatin	Microglia	Minocycline	[111]
CCL1/CCR8	Bortezomib	Astrocyte	‐	[35]
PK2	Bortezomib	Both	PC1 (a prokineticin receptor antagonist)	[112]
P2X7R/p38 MAPK	Bortezomib	Microglia	‐	[113]

Abbreviations: ALA, alpha‐lipoic acid; AIBP, apolipoprotein A‐I binding protein; C5aR1, complement component 5a receptor 1; C/EBP‐β, CCAAT/enhancer‐binding protein beta; CCL, C‐C motif chemokine ligand; CCR, C‐C motif chemokine receptor; LPS‐RS, lipopolysaccharide from Rhodobacter sphaeroides; l‐CDL, levo‐corydalmine; IL‐1ra, interleukin‐1 receptor antagonist; MAPK, mitogen‐activated protein kinase; MLKL, mixed lineage kinase domain‐like protein; NAC, n‐acetylcysteine; NF‐κB, nuclear factor kappaB; PEA‐OXA, palmitoylethanolamide‐oxazoline; PGE2, prostaglandin E2; RIP3, receptor interacting‐protein 3; S1P, sphingosine‐1‐phosphate; TRPV1, transient receptor potential vanilloid 1; EP2, E‐prostanoid receptor 2; TGF‐β1, transforming growth factor beta 1; P2Y12, P2Y purinoceptor 12; IL‐18, lnterleukin‐18; TLR4, Toll‐like receptor 4; TREM2, triggering receptor expressed on myeloid cells 2; DAP12, DNAX‐activating protein of 12 kDa; PK2, prokineticin 2; P2X7R, P2X7 receptor.

### 4.1 Paclitaxel

Paclitaxel, a taxane chemotherapeutic agent derived from the yew tree and has been approved by the FDA for the treatment of various solid tumors.^114^ Its antitumor activity is primarily achieved by stabilizing microtubules and inducing apoptosis, thereby arresting the cell cycle.^115^, ^116^ However, paclitaxel commonly induces painful peripheral neuropathy, which represents one of its major adverse effects.

#### 4.1.1 Astrocyte

Paclitaxel can induce marked and persistent hyperalgesia. Concurrently, it increases the expression of glial fibrillary acidic protein (GFAP, an astrocytic marker) and OX‐42 (a monoclonal antibody against CD11b, used as a microglial marker) in the spinal dorsal horn, accompanied by elevated levels of various pro‐inflammatory cytokines and chemokines in this region, without evidence of neuronal damage.^117^ Reactive astrocytes can be classified into A1 and A2 phenotypes according to their functions. After neural injury, A1 astrocytes secrete neurotoxins, whereas A2 astrocytes exert neuroprotective and reparative effects.^40^ Reports have shown that paclitaxel promotes the transformation of astrocytes into the A1 phenotype, whereas inhibition of the Notch signaling pathway attenuates this transformation and alleviates paclitaxel‐induced pain hypersensitivity. With repeated administrations, the number of A1 astrocytes progressively increases, suggesting their contribution to pathological processes during the maintenance phase.^104^ Multiple studies indicate that Toll‐like receptor 4 (TLR4) is a key receptor mediating paclitaxel‐induced glial activation. An early study by Li et al. reported increased TLR4 expression in spinal dorsal horn astrocytes after paclitaxel administration, suggesting that paclitaxel may activate astrocytes through TLR4 binding, whereas no such increase was observed in microglia.^65^ However, Moraes et al. recently showed that, at an early stage, paclitaxel induces injured primary sensory neurons or glial cells to release high‐mobility group box 1 (HMGB1). This molecule activates TLR4 on spinal microglia and initiates downstream nuclear factor kappaB (NF‐κB) and mitogen‐activated protein kinase (MAPK) signaling, leading to elevated pro‐inflammatory cytokines that contribute to pain.^42^ This mechanism has been further validated in the studies by Shi et al. and Santos et al.^66^, ^81^ In addition, paclitaxel has been reported to directly bind to and activate complement component 5a receptor 1 (C5aR1). This activation triggers glial responses, resulting in the release of inflammatory mediators such as CCL2, IL‐1β, and tumor necrosis factor (TNF)‐α, and upregulates membrane ion channels in sensory neurons, including transient receptor potential ankyrin subtype 1 protein, ultimately contributing to hyperalgesia.^106^ Consistently, another study demonstrated that paclitaxel induces glial activation in the spinal dorsal horn, accompanied by increased transient receptor potential vanilloid 1 (TRPV1) expression.^101^ Furthermore, evidence indicates that electroacupuncture alleviates paclitaxel‐induced mechanical allodynia by suppressing astrocytic and microglial activation, primarily through inhibition of the TLR4/NF‐κB pathway and TRPV1 channels.^67^, ^68^ Recently, Chen et al. further reported that electroacupuncture can suppress astrocytic activation in the rostral ventromedial medulla (RVM) and inhibit their calcium signaling activity, thereby mitigating paclitaxel‐induced mechanical hyperalgesia.^118^

Abnormal activation of astrocytes can also indirectly disrupt glutamatergic synaptic homeostasis. At an early stage, paclitaxel rapidly reduces the expression of the glutamate transporters GLAST and GLT‐1 in spinal dorsal horn astrocytes.^95^ Mechanistically, paclitaxel activates spinal nicotinamide adenine dinucleotide phosphate oxidase, which in turn partially inactivates the astrocytic glutamate transporter GLT‐1 and the enzyme glutamine synthetase. This impairment diminishes glutamate clearance, leading to its accumulation in the synaptic cleft, thereby enhancing neuronal excitability and aggravating central sensitization.^96^, ^119^ In addition, Gao et al. demonstrated that inhibiting glycogen synthase kinase 3β significantly reduces GFAP expression in the spinal dorsal horn and restores GLT‐1 levels, thereby alleviating paclitaxel‐induced hyperalgesia.^97^ Notably, research on the role of glutamate transporters in CIPN has been relatively limited in recent years. Reactive changes in astrocytes also involve the secretion of factors that modulate excitatory synapses. For instance, paclitaxel can induce astrocytic secretion of SPARC Like 1 (SPARCL1), which promotes the formation of excitatory synapses in the spinal cord and contributes to the development of neuropathic pain; this effect can be inhibited by a sphingosine‐1‐phosphate receptor 1 (S1PR1) antagonist.^99^

#### 4.1.2 Microglia

Some early studies questioned whether microglia are activated,^65^, ^95^ but growing evidence supports a critical role for microglia in paclitaxel‐induced peripheral neuropathy.^107^, ^108^, ^120^, ^121^ For instance, Makker et al. have shown that expression of purinergic receptor P2Y12 (P2RY12), a homeostatic microglial marker, is reduced in the spinal dorsal horn after paclitaxel treatment, indicating a shift toward an activated state.^121^ Similar to astrocytes, reactive microglia can also be classified into two phenotypes: M1 microglia exhibit cytotoxic properties, whereas M2 microglia are associated with neuroprotective and reparative functions.^122^ Ma et al. further demonstrated that paclitaxel induces M1 polarization of microglia, promotes the release of inflammatory cytokines, and activates the RIP3/MLKL‐mediated necroptosis pathway, thereby aggravating neuronal injury and pain symptoms. Minocycline attenuates neuronal necroptosis and alleviates hyperalgesia by inhibiting M1 polarization, suggesting that M1 polarization contributes to the nociceptive process.^107^ Moreover, Guo et al. reported that microglia can also interact with neurons via the PGE₂–EP2 pathway, providing an additional mechanism contributing to hyperalgesia.^108^ Another study demonstrated that paclitaxel induces dysfunction of spinal dorsal horn microglia and upregulates brain‐derived neurotrophic factor (BDNF) expression. These changes lead to neuronal sensitization and mechanical hyperalgesia, an effect reversible by a cannabinoid receptor type 2 (CB₂) receptor agonist.^123^ Similarly, Meng et al. found that hyperbaric oxygen suppresses paclitaxel‐induced upregulation of spinal GFAP and CD11b, and this effect also depends on activation of spinal CB₂ receptors.^82^ In addition, Toma et al. reported that an α7 nicotinic acetylcholine receptors (nAChR) silent agonist alleviates paclitaxel‐induced hyperalgesia and suppresses abnormal microglial activation.^124^ Micheli et al. also demonstrated that the non‐opioid analgesic DDD‐028 alleviates pain via the α7 nAChR pathway while concurrently inhibiting microglial and astrocytic activation in the spinal cord and primary somatosensory cortex.^125^ By contrast, other studies have reported that paclitaxel selectively activates astrocytes in the primary somatosensory cortex without significantly affecting spinal glial cells. Despite discrepancies across studies, these findings suggest that cortical glial activation may also contribute to the development of CIPN.^126^

### 4.2 Vincristine

Vincristine is a vinca alkaloid chemotherapeutic agent used in the treatment of multiple malignancies, including neuroblastoma, cervical cancer, lymphoma, and breast cancer. However, it exhibits pronounced neurotoxicity. When the cumulative dose exceeds 4 mg/m², patients may develop nerve injury of varying severity,^127^ with the incidence reaching up to 78% in adults.^128^, ^129^

#### 4.2.1 Astrocyte

Astrocytes are key contributors to vincristine‐induced peripheral neuropathy. An early study by Ji et al. demonstrated that vincristine treatment in rats induced mechanical hyperalgesia accompanied by spinal astrocyte activation. Activated astrocytes release IL‐1β, which induces phosphorylation of NMDA receptors in dorsal horn neurons, thereby enhancing pain transmission. The study also indicated that oxidative stress is an important trigger for astrocyte activation.^84^ The astrocyte‐mediated inflammatory response is critical in this process. Mechanistically, Hu et al. revealed that the Wnt/β‐catenin pathway drives vincristine‐induced glial inflammation and spinal central sensitization by modulating astrocyte and microglial activation.^41^ More recently, Chen et al. reported that vincristine drives spinal astrocytes toward a pro‐inflammatory A1 phenotype through the C/EBP‐β/TGF‐β1 signaling pathway, without affecting A2 polarization.^109^ Furthermore, Li et al. demonstrated the involvement of T‐type calcium channel Cav3.2 and Ca2^+^/calmodulin‐dependent protein kinase II (CaMKII) in astrocyte activation. Vincristine activates CaMKII and CaV3.2, thereby increasing intracellular calcium levels in astrocytes, which in turn promotes connexin43–mediated release of inflammatory cytokines and ultimately leads to central sensitization.^130^ Their subsequent research further showed that the CaMKII/NF‐κB pathway mediates connexin43–dependent inflammation, oxidative stress, and apoptosis, whereas celastrol, a potential therapeutic agent, suppresses astrocyte activation and alleviates aberrant pain.^19^

NF‐κB is recognized as a key regulator of astrocyte‐mediated neuroinflammation. For instance, activation of NF‐κB in spinal dorsal horn astrocytes can drive the synthesis and release of the chemokine CXCL1, which binds to C‐X‐C motif chemokine receptor 2 (CXCR2) receptors on neighboring neurons and thereby enhances the excitability of central pain pathways.^131^ Similarly, Zhou et al. reported that vincristine upregulates spinal CXCL1 and its receptor CXCR2, accompanied by NF‐κB pathway activation. Functional studies further showed that levo‐corydalmine suppresses NF‐κB activation, reducing astrocytic CXCL1 release and alleviating hyperalgesia. However, this inhibitory effect is abolished when NF‐κB is silenced.^69^ Collectively, these findings suggest that targeting the astrocytic NF‐κB pathway may represent an effective strategy for alleviating CIPN. Notably, the NF‐κB and nuclear factor erythroid‐2‐related factor 2 (Nrf2) pathways mutually inhibit each other.^132^ Nrf2, a central regulator of the cellular antioxidant response, induces the expression of antioxidant enzymes such as heme oxygenase‐1 (HO‐1) to counteract oxidative damage.^132^, ^133^ In a subsequent study, Zhou et al. reported that vincristine inhibits the Nrf2 pathway, leading to reduced HO‐1 expression and carbon monoxide (CO) production, thereby enhancing TNF‐α–induced connexin43 expression and hemichannel activity. Using HO‐1 inducers, inhibitors, and gene silencing, they confirmed that vincristine‐induced astrocytic connexin43 activation is mediated through the Nrf2/HO‐1/CO signaling pathway.^134^ This result partially aligns with the findings of Li et al.^19^ Shen et al. similarly observed activation of spinal dorsal horn astrocytes and microglia, together with increased HO‐1 expression, in a vincristine mouse model. These changes were accompanied by MAPK pathway activation and the release of multiple inflammatory factors. Exogenous induction of HO‐1 effectively suppressed abnormal glial activation and inflammatory factor release, thereby alleviating CIPN symptoms.^78^

#### 4.2.2 Microglia

The contribution of microglia has also attracted increasing attention. Qin et al. demonstrated that vincristine significantly upregulates the chemokine C‐X3‐C motif chemokine ligand 1 (CX3CL1) and its receptor CX3CR1 in the spinal cord, which in turn activates microglia and triggers the downstream p38 MAPK signaling pathway. This cascade promotes the release of inflammatory cytokines, ultimately leading to CIPN.^77^ Further studies showed that inhibiting the upstream Notch pathway significantly reduces CX3CR1 and phosphorylated p38 expression in microglia.^105^ Additionally, Shao et al. reported that vincristine induces microglial polarization toward the pro‐inflammatory M1 phenotype and promotes inflammatory cytokine release via the NF‐κB pathway. Conversely, inhibition of this pathway drives polarization toward the anti‐inflammatory phenotype and alleviates pain symptoms.^70^ This study provides the first evidence implicating the NF‐κB pathway in vincristine‐induced microglial activation and hyperalgesia. Moreover, in line with the findings of Chen et al.,^109^ vincristine was shown to induce glial phenotypic transformation through distinct mechanisms, thereby contributing to the onset and progression of CIPN. It is also noteworthy that El‐Sawaf et al. also observed concurrent abnormal activation of spinal microglia and astrocytes following vincristine administration, accompanied by the release of multiple inflammatory cytokines. This inflammatory response was attenuated by melatonin, which alleviated hyperalgesia.^135^

### 4.3 Platinum‐based chemotherapeutic agents

Platinum‐based chemotherapeutic agents, such as cisplatin and oxaliplatin, are first‐line treatments for a variety of solid tumors, including ovarian, head and neck, testicular, and bladder cancers.^136^ The neurotoxicity of cisplatin, a first‐generation platinum compound, has been extensively reported.^137^ Although the third‐generation platinum drug oxaliplatin offers improved antitumor efficacy, its neurotoxicity has not been substantially reduced.^138^, ^139^

#### 4.3.1 Oxaliplatin

##### Astrocyte

In oxaliplatin‐induced painful peripheral neuropathy, astrocytes contribute to both the initiation and maintenance of pain through inflammatory responses and glia–neuron interactions.^71^, ^72^, ^85^, ^140^, ^141^, ^142^ Man et al. found that oxaliplatin upregulates GFAP expression in the central nervous system and activates the TLR4/NF‐κB pathway, thereby promoting the release of pro‐inflammatory cytokines such as TNF‐α.^71^ Further evidence was provided by Campolo et al., who showed that in the oxaliplatin model, spinal dorsal horn astrocytes are markedly activated, accompanied by a specific increase in IL‐17, while IL‐1β and TNF‐α expression is also significantly upregulated, an effect associated with NF‐κB pathway activation. Notably, the endogenous antioxidant transcription factor Nrf2 and its downstream antioxidant enzymes are not effectively activated.^72^ In addition to inflammatory mechanisms, oxaliplatin can also directly damages astrocytes. For example, Cinci et al. demonstrated that 100 µM oxaliplatin significantly increases superoxide anion production in primary rat astrocytes and induces oxidative stress while activating caspase‐3, indicating apoptosis‐like injury in these cells.^86^ In in vivo models, the astrocytic response appears to be stage‐dependent. For example, an early study by Kim et al. reported that oxaliplatin can induce upregulation of ionized calcium‐binding adaptor molecule 1 (IBA‐1) expression and oxidative stress in the spinal cord, but no obvious changes in GFAP are observed.^143^ In contrast, Janes et al. observed only significant activation of astrocytes in the spinal dorsal horn.^91^ Recently, Melato et al. demonstrated that acute oxaliplatin administration rapidly induces microglial activation and upregulates IL‐1β, TNF‐α, and BDNF, whereas GFAP immunoreactivity of astrocytes remains unchanged during the same period.^144^ These discrepancies may be related to differences in oxaliplatin dosing regimens and observation windows.

Inhibition of inflammatory pathways and oxidative stress can alleviate oxaliplatin‐induced hyperalgesia. A previous study demonstrated that blocking the COX‐2/NF‐κB pathway and reducing oxidative stress significantly attenuate astrocyte activation and downregulate COX‐2 expression along with multiple pro‐inflammatory cytokines.^73^ Dong et al. further elucidated the roles of inflammation and oxidative stress: on one hand, astrocyte activation markedly increases TNF‐α, IL‐1β, and NF‐κB signaling in the spinal cord^87^; on the other hand, oxaliplatin induces mitochondrial dysfunction in the spinal cord, leading to elevated reactive oxygen species (ROS) levels and activation of the NLR family pyrin domain containing 3 (NLRP3) inflammasome.^85^ These two pathological processes interact and jointly contribute to oxaliplatin‐induced peripheral neuropathy. Similarly, Agnes et al. and Areti et al. also confirmed that oxaliplatin impairs mitochondrial function in the spinal cord and increases ROS release, accompanied by pronounced astrocyte activation and pro‐inflammatory cytokine release.^88^, ^89^ In addition, rosmarinic acid has been shown to suppress abnormal astrocyte activation and improve mitochondrial function, thereby alleviating oxaliplatin‐induced pain behaviors.^89^ Multiple studies have shown that the astrocytic adenosine signaling pathway plays an important regulatory role in oxaliplatin‐induced peripheral neuropathy. Wahlman et al. reported that oxaliplatin upregulates cytoplasmic adenosine kinase (ADK) expression in astrocytes, thereby attenuating the antinociceptive effect of adenosine mediated by A3 adenosine receptors (A3AR). At the same time, the spinal NLRP3/IL‐1β pathway is activated by oxaliplatin, whereas an A3AR agonist inhibits this pathway and promotes the expression of the anti‐inflammatory cytokine IL‐10.^92^ Consistent with these findings, Janes et al. observed that selective activation of A3AR inhibits abnormal astrocyte activation. This intervention also reduces the production of pro‐inflammatory cytokines and increases anti‐inflammatory cytokine levels, thereby alleviating oxaliplatin‐induced hyperalgesia.^91^ Another study demonstrated that specific restoration of A1 adenosine receptors on spinal astrocytes via a lentiviral vector increases the levels of glutamate metabolism‐related proteins and effectively alleviates pain symptoms.^145^

Current research indicates that astrocytes, through interactions with neurons and various signaling molecules, play a significant regulatory role in the development and persistence of CIPN. For example, Le et al. demonstrated that oxaliplatin reduces the expression of insulin‐like growth factor‐1 (IGF‐1) originating from spinal dorsal horn astrocytes, whose receptors are primarily located on adjacent neurons. Reduced IGF‐1 levels increase the expression of the pro‐nociceptive mediators IL‐17A and calcitonin gene‐related peptide (CGRP), whereas supplementation with recombinant IGF‐1 significantly alleviates oxaliplatin‐induced mechanical hyperalgesia.^20^ Another example of glia–neuron communication is vascular endothelial growth factor A (VEGF‐A). Micheli et al. reported that oxaliplatin markedly upregulates VEGF‐A expression in spinal dorsal horn astrocytes and activates neuronal VEGFR‐1 receptors, thereby promoting the release of pain‐related mediators. Selective knockdown of VEGF‐A effectively alleviates oxaliplatin‐induced hyperalgesia, suggesting that astrocytes and neurons may amplify pain signaling through the VEGF‐A/VEGFR‐1 pathway.^146^, ^147^ Interestingly, VEGF‐A exerts dual effects. Toti et al. recently reported that astrocyte‐derived VEGF‐A can also confer neuroprotection by activating neuronal VEGFR‐2 receptors, thereby suppressing excessive astrocyte activation and neuronal injury.^147^ Consistently, under specific signaling conditions, astrocytes may also exert protective effects. For instance, oxaliplatin induces spinal neurons to release IL‐1α, which acts on astrocytes to stimulate the release of transforming growth factor‐β1 (TGF‐β1) and reduce the accumulation of extracellular ATP, thereby mitigating neuronal damage. Intrathecal injection of IL‐1α increases astrocyte numbers in the spinal cord and effectively reverses oxaliplatin‐induced hyperalgesia.^148^ Another study showed that oxaliplatin upregulates pituitary adenylyl cyclase‐activating polypeptide and activates spinal PAC1 receptors, thereby causing pronounced astrocyte activation. Notably, blockade of PAC1 receptors effectively alleviates oxaliplatin‐induced cold allodynia.^149^ Similarly, ion channels in sensory neurons are also involved in regulating astrocytes. Lee et al. found that a single intraperitoneal injection of oxaliplatin induces pronounced cold allodynia in mice, accompanied by increased expression of TRPV1 and GFAP in the spinal dorsal horn. Administration of a TRPV1 antagonist not only suppresses TRPV1 overexpression but also markedly reduces GFAP levels, thereby alleviating abnormal pain responses.^102^, ^103^

##### Microglia

Multiple studies have reported increased levels of microglia‐derived cytokines in oxaliplatin models. For example, Yang et al. showed that spinal microglia are markedly activated by oxaliplatin, accompanied by significant increases in IL‐1β and monocyte chemoattractant protein‐1.^150^ Inflammatory mediators released by microglia contribute directly to pain sensitization. In another study, Lee et al. reported that oxaliplatin treatment induces a marked increase in IBA‐1‐labeled microglia within the spinal dorsal horn and enhances phosphorylation of extracellular signal‐regulated kinase (ERK) and NF‐κB, thereby triggering an inflammatory cascade that results in cold and mechanical allodynia.^79^ In addition, it was reported that activation of microglia is accompanied by elevated expression of the pain‐related markers c‐Fos and CGRP. Mechanistically, oxaliplatin induces aberrant microglial activation by suppressing the AMPK–SOCS3 signaling axis and upregulating the TLR4/p38 MAPK pathway, thereby amplifying pain signaling.^43^ Pyroptosis of microglia represents another mechanism underlying oxaliplatin‐induced pain. Chen et al. reported that the lysine acetyltransferase 2A (KAT2A) drives microglial pyroptosis by promoting succinylation of p38 MAPK. Pharmacological inhibition of the KAT2A–p38 axis markedly attenuates spinal inflammation and pain‐like behaviors, whereas overexpression of KAT2A counteracts this analgesic effect.^151^

There is also a close interplay between oxidative stress and microglial activation. Evidence shows that oxaliplatin‐induced microglial activation is accompanied by elevated ROS levels and the release of mitochondrial cytochrome c in spinal tissues. This suggests that oxaliplatin may promote microglial activation by inducing oxidative stress and endoplasmic reticulum stress, thereby contributing to neurotoxicity and pain abnormalities.^90^ Another study further supports this notion, demonstrating that an adenosine A2A receptor antagonist reduces microglial activity and oxidative stress in the oxaliplatin model, thereby alleviating neuropathic pain symptoms.^93^ Together with findings from astrocytes, these results suggest that targeting adenosine receptors may represent an effective approach for alleviating CIPN. Recently, the role of the α7 nAChR in alleviating oxaliplatin‐induced neuroinflammation and pain has attracted attention. Marmouzi et al. reported that oxaliplatin induces abnormal activation of spinal microglia and astrocytes, accompanied by activation of the TLR4/NF‐κB pathway, upregulation of pro‐inflammatory factors, and increased expression of pro‐brain‐derived neurotrophic factor. Activation of α7 nAChR markedly suppressed the above alterations, thereby alleviating oxaliplatin‐induced pain hypersensitivity.^74^ Emerging strategies also aim to simultaneously modulate the functions of both astrocytes and microglia to relieve pain. For example, Wang et al. found that overexpression of mesencephalic astrocyte‐derived neurotrophic factor significantly reduces the number of activated glial cells in the spinal cord and suppresses the activation of key inflammatory pathways such as NF‐κB and ERK, thereby attenuating oxaliplatin‐induced mechanical allodynia.^152^ These findings suggest that enhancing the neurotrophic support of glial cells or inhibiting their inflammatory responses may serve as effective strategies for alleviating CIPN.

#### 4.3.2 Cisplatin

##### Astrocyte

In vitro studies by Jiang et al. demonstrated that low‐dose cisplatin (12.5 μM) inhibits the proliferation of primary rat astrocytes and induces delayed cell death, accompanied by the sustained downregulation of several autophagy‐related proteins, particularly LC3‐II. In contrast, 6.25 μM cisplatin only inhibits proliferation without inducing cell death, suggesting a dose‐dependent toxicity of cisplatin toward astrocytes.^153^ Leo et al. further observed that after 24 h of cisplatin treatment, spinal astrocytes are markedly activated, accompanied by downregulation of the astrocytic inward rectifier potassium channel 4.1 (Kir4.1) and the glutamate transporter excitatory amino acid transporter 1 (EAAT1). Dysfunction of Kir4.1 impairs the astrocytic clearance of perisynaptic K⁺, which in turn suppresses EAAT1‐mediated glutamate uptake, leading to extrasynaptic glutamate accumulation and neuronal hyperexcitability.^98^ These findings suggest that cisplatin may promote central sensitization by disrupting ionic homeostasis and neurotransmitter clearance in astrocytes. Sustained cisplatin administration can disrupt the blood–brain barrier.^154^ In a cisplatin‐induced CIPN model, Wang et al. observed elevated GFAP and S100β levels in the periaqueductal gray (PAG) region, accompanied by morphological alterations of astrocytes. Specifically, astrocytic processes appear more extended in two‐dimensional views, whereas three‐dimensional structural analysis reveals a reduction in overall cell volume, consistent with a typical activated state. Moreover, signals released from central neurons can further amplify astrocytic responses.^155^ Xie et al. reported that cisplatin induces upregulation of CGRP in the spinal cord, which activates astrocytes. Subsequent in vitro experiments confirmed that CGRP promotes both astrocytes and microglia to release multiple pro‐inflammatory factors and mediates interglial inflammatory signaling through the CCL3/CCR3 pathway, thereby forming a cascade that amplifies glial inflammatory responses.^36^ In addition to inflammatory mechanisms, cisplatin also affects glial cells through metabolic stress pathways. Singh et al. reported that cisplatin induces mechanical allodynia and spontaneous pain in mice, accompanied by upregulated A3AR expression in both microglia and astrocytes. This observation is consistent with findings described for oxaliplatin in this review.^91^, ^92^, ^94^ Moreover, cisplatin induces synaptosomal mitochondrial dysfunction and oxidative stress. Administration of the A3AR agonist MRS5980 not only alleviates cisplatin‐induced allodynia but also confers neuroprotection.^94^ Although A3AR agonists exhibit certain anti‐inflammatory potential, this study did not detect typical glial inflammatory responses, suggesting that metabolic stress and mitochondrial damage may represent one of the key mechanisms underlying cisplatin‐induced pain.

##### Microglia

Microglia‐mediated neuroinflammation is a key process in cisplatin‐induced peripheral neuropathy. In mice administered cisplatin, Kim et al. observed mechanical and cold allodynia, together with elevated TLR4 expression in microglia and astrocytes and significant increases in the downstream pro‐inflammatory cytokines TNF‐α and IL‐1β.^83^ Woller et al. further confirmed that this allodynia depends on TLR4 pathway activation, with microglia serving as the primary effector cells.^75^ Inhibition of the endocannabinoid‐metabolizing enzyme by JZL195 downregulates TLR4 expression in glial cells, attenuates the inflammatory response, and alleviates allodynia.^83^ In addition to the TLR4 pathway, cisplatin can also induce pro‐inflammatory activation of microglia through alternative signaling routes. For example, Chen et al. demonstrated that cisplatin upregulates P2Y12 receptors in spinal microglia. Activation of this receptor promotes microglial release of IL‐18 via Src kinase and p38 MAPK pathways, thereby driving central sensitization and pain amplification. Blocking P2Y12 markedly attenuates pain hypersensitivity and aberrant microglial activation.^110^ Another study revealed that cisplatin drives microglial polarization toward the pro‐inflammatory M1 phenotype through the triggering receptor expressed on myeloid cells 2 (TREM2)/DNAX‐activating protein of 12 kDa (DAP12) pathway, accompanied by upregulation of multiple inflammatory factors. Blocking TREM2 alleviates cisplatin‐induced allodynia and neuronal injury, underscoring the critical role of microglia‐mediated neuroinflammation in CIPN.^111^ Consistent with these findings, Ma et al. further found that cisplatin downregulates G protein‐coupled receptor kinase 2 (GRK2) in spinal dorsal horn neurons. Loss of neuronal GRK2 exacerbates excessive activation of the microglial TREM2/DAP12 pathway, whereas GRK2 overexpression suppresses this pathway, reduces inflammatory mediator release, and thereby alleviates CIPN.^156^ In this neuron–microglia interaction, microRNAs (miR) have also been identified as critical regulators. Li et al. found that cisplatin downregulates spinal neuronal miR‐124, which under physiological conditions restrains microglial activation. Intrathecal administration of an miR‐124 mimic suppresses aberrant microglial activation and inflammatory mediators upregulation, thereby alleviating mechanical allodynia. This finding indicates that the loss of miR‐124 is a key driver of pro‐inflammatory microglial polarization.^17^

One study highlighted the axis linking the gut microbiota to central glial immune responses: cisplatin can increase blood–spinal cord barrier permeability, leading to the accumulation of inflammatory factors in the spinal cord and microglial activation via the IL‐1β/NF‐κB pathway.^157^ This inflammatory mechanism has also been confirmed in the peripheral nerves.^158^ Interestingly, fecal microbiota transplantation corrects cisplatin‐induced gut dysbiosis and restores blood–spinal cord barrier function. This intervention also suppresses NF‐κB signaling and microglial activation, ultimately alleviating pain symptoms.^157^, ^159^ These results provide a novel therapeutic perspective for CIPN, although efficacy requires further validation. Zhang et al. further investigated microglial activation from an epigenetic perspective. They showed that cisplatin disrupts epigenetic homeostasis in microglia by downregulating key histone methylation marks (such as H3.1K27me1 and H3K56me3), thereby inducing microglial activation and promoting pain behaviors. Targeting the histone lysine demethylase 7A (KDM7A) provides a novel avenue for the epigenetic therapy of CIPN, although its translational value in cancer patients remains uncertain.^160^ In addition, researchers employed the bivalent opioid receptor agonist MCC22 to attenuate cisplatin‐induced mechanical allodynia by inhibiting microglial activation,^161^ suggesting that modulation of opioid receptors may represent a potential strategy for the prevention and treatment of CIPN.

### 4.4 Bortezomib

Bortezomib is the first proteasome inhibitor to enter clinical use and has demonstrated remarkable efficacy as a first‐line chemotherapeutic agent for multiple myeloma and mantle cell lymphoma.^162^, ^163^ However, bortezomib also exhibits considerable neurotoxicity, damaging nerve fibers through multiple pathways and inducing peripheral neuropathy.^164^

#### 4.4.1 Astrocyte

Robinson et al. first reported that bortezomib can induce mechanical allodynia in rats, closely associated with astrocytic activation. GFAP expression in the spinal dorsal horn is significantly increased on Days 7, 14, and 30 after administration, in parallel with the time course of allodynia. Treatment with minocycline blocked the GFAP upregulation and attenuated allodynia, suggesting that astrocyte activation mediates bortezomib‐induced pain abnormalities through inflammatory mechanisms.^165^ However, this study did not examine inflammatory markers for validation, although subsequent research has confirmed this association. For example, Yardim et al. reported that bortezomib induces astrocyte activation accompanied by upregulation of TNF‐α, IL‐1β, and IL‐6. They further observed the activation of multiple pro‐inflammatory signaling pathways, including NF‐κB, TLR4, and NLRP3.^76^ Another study showed that following bortezomib treatment, IL‐1β expression is significantly increased in astrocytes, whereas TNF‐α is predominantly expressed in neurons.^80^ Notably, selective blockade of the neuronal NF‐κB pathway significantly alleviates bortezomib‐induced allodynia in mice.^166^ Astrocyte–neuron interactions are crucial for regulating inflammatory signaling in CIPN. Recently, Chen et al. further validated this effect, demonstrating that bortezomib‐induced mechanical allodynia appears earlier in female mice, consistent with clinical observations. Mechanistically, perivascular macrophages infiltrate the spinal perivascular region of female mice at an early stage and release CCL1, which activates astrocytes via its receptor CCR8 and triggers calcium signaling, ultimately leading to CIPN through glia–neuron interactions.^35^

Other signaling mechanisms related to astrocytes are also implicated in bortezomib‐induced allodynia. Suzuki et al. demonstrated that intrathecal administration of a mammalian target of rapamycin (mTOR) inhibitor alleviates bortezomib‐induced mechanical allodynia. However, its inhibitory effect on spinal astrocyte activation showed only a downward trend without reaching statistical significance, suggesting that the analgesic effect of mTOR pathway inhibition may not be limited to the regulation of astrocyte activation.^167^ Meanwhile, Stockstill et al. first identified the role of sphingolipid metabolic dysregulation in bortezomib‐induced peripheral neuropathy. They found that bortezomib increases sphingosine‐1‐phosphate (S1P) levels in the spinal dorsal horn and activates its receptor S1PR1, thereby initiating a neuroinflammatory response primarily mediated by astrocytes and ultimately disrupting glutamate homeostasis.^100^ A follow‐up study by Robinson et al. further supplemented this observation, showing that bortezomib upregulates connexin43 and downregulates GLAST in spinal astrocytes, jointly leading to disruption of glutamate homeostasis and thereby maintaining central sensitization.^44^ Pharmacological inhibition of S1PR1 dose‐dependently alleviates mechanical allodynia. In contrast, mice with astrocyte‐specific S1PR1 deletion neither develop pain behaviors nor respond to the S1PR1 antagonist. Such evidence further confirms the mediating role of the astrocytic S1P/S1PR1 pathway in bortezomib‐induced peripheral neuropathy.^100^ In addition, Moschetti et al. observed that after 14 days of bortezomib treatment, GFAP expression is markedly upregulated in the mouse spinal cord. By day 28, prokineticin 2 (PK2) and its receptor are upregulated in astrocytes, accompanied by increased expression of the microglial markers CD68 and TLR4 and associated inflammatory responses. The prokineticin receptor antagonist PC1 suppresses these pathological changes and alleviates abnormal pain in mice.^112^ Another study also confirmed the regulatory role of the prokineticin system in bortezomib‐induced glial inflammatory responses and pain in higher central regions, including the hippocampus, prefrontal cortex, and hypothalamus. Collectively, these findings suggest that the prokineticin system may mediate painful CIPN through coordinated actions in the spinal cord and higher centers of the ascending pathway.^168^

#### 4.4.2 Microglia

Although some controversy remains regarding the role of microglia in bortezomib‐induced peripheral neuropathy,^35^, ^165^, ^167^ multiple studies have confirmed that their activation is a key contributor to this process.^112^, ^113^, ^169^ Unlike the aforementioned chemotherapeutic agents, bortezomib‐induced microglial activation appears to occur at a later stage of administration.^112^ Guo et al. reported that the P2X7 receptor (P2X7R) is primarily expressed in activated microglia in the spinal dorsal horn, and its expression is significantly upregulated following bortezomib treatment, accompanied by enhanced phosphorylation of p38 MAPK. Functional experiments further showed that a P2X7R antagonist suppresses excessive phosphorylation of p38 in microglia and downregulates the mRNA levels of inflammatory factors, including IL‐1β, IL‐6, and TNF‐α, thereby markedly alleviating bortezomib‐induced mechanical allodynia. Similarly, a p38 inhibitor decreases inflammatory cytokine expression and suppresses the upregulation of P2X7R in microglia, suggesting a bidirectional regulatory relationship between P2X7R and p38 MAPK. In summary, the microglial P2X7R/p38 MAPK signaling axis synergistically mediates bortezomib‐induced painful peripheral neuropathy and may serve as a potential therapeutic target.^113^ However, another study reported that bortezomib significantly enhances the phosphorylation of c‐Jun N‐terminal kinase (JNK) in astrocytes, but unlike the findings of Guo et al., this study did not find a significant change in p38 MAPK phosphorylation.^80^ The reported differences suggest that the roles of MAPK family pathways may differ across glial cell types. Nevertheless, the overall evidence indicates that the MAPK signaling family plays a critical role in bortezomib‐induced neuropathy, with its pro‐inflammatory effects largely mediated by glial activation.^80^, ^113^ In addition to intrinsic glial signaling pathways, bortezomib can indirectly activate microglia via vascular‐derived factors. Studies have shown that bortezomib does not compromise the integrity of the blood–brain barrier but induces IL‐23A upregulation and secretion in brain microvascular endothelial cells. Endothelium‐derived IL‐23A binds to IL‐23 receptors on microglia, inducing their activation and thereby triggering inflammatory responses in the central nervous system.^169^

## 5 OTHER TYPES OF GLIAL CELLS IN CIPN

Beyond microglia and astrocytes, other types of glial cells may also contribute to the development and persistence of CIPN. Oligodendrocytes represent a major glial cell subtype that provides axonal conduction and metabolic support, and their dysfunction or myelin abnormalities may enhance nociceptive signaling within the spinal cord.^170^, ^171^ Previous studies have reported that cisplatin treatment in mice increases adenosine A3 receptor expression in glial cells, with oligodendrocytes showing the most prominent changes. Administration of an A3AR agonist reverses cisplatin‐induced neurotoxicity.^94^ In contrast, another study did not detect alterations in oligodendrocytes within the CIPN model.^111^ Although direct evidence in CIPN remains limited, related findings provide some support. For instance, cisplatin has been shown to induce central white matter and myelin abnormalities in mice, which can be restored by bexarotene, thereby normalizing myelin‐associated pathways and motor–sensory function.^172^ Recent studies and single‐cell analyses suggest that, under neuropathic pain and chemotherapy‐related conditions, oligodendrocyte and myelin damage, inflammatory responses of oligodendrocyte lineage cells, and remyelination remodeling may contribute to the initiation and persistence of central sensitization and pain plasticity.^170^, ^173^ Nevertheless, the available evidence remains limited, and their precise roles in the pathogenesis of CIPN require further clarification.

Satellite glial cells (SGCs) closely ensheath the somata of the dorsal root ganglion (DRG) neurons and regulate their excitability.^174^ In CIPN, SGCs exhibit phenotypic alterations, including gap junction remodeling, enhanced purinergic signaling, and activation of innate immune receptors. These changes facilitate neuron–glia crosstalk and increase the excitability of primary afferent neurons.^175^, ^176^, ^177^ Previous studies have shown that SGC activation contributes to paclitaxel‐ and platinum‐induced neuropathies,^178^ and more recent work has identified the TLR9 signaling pathway in SGCs as a driver of paclitaxel‐induced mechanical allodynia.^179^ Moreover, single‐cell RNA sequencing has been employed to resolve SGC heterogeneity and transcriptional programs in CIPN models.^180^ Anatomically, the DRG is located outside the classical blood–brain barrier, a characteristic that enhances the translational feasibility of SGC‐targeted interventions, such as modulation of purinergic/TLR receptors or the use of connexin channel inhibitors.^174^, ^181^

## 6 CONCLUSION AND PERSPECTIVES

A growing body of evidence has shown that spinal microglia and astrocytes play an important role in the development of CIPN and have preliminarily elucidated multiple potential molecular mechanisms. Multiple chemotherapeutic agents can induce abnormal activation of microglia and astrocytes, causing persistent neuroinflammation that enhances pain signal transmission and leads to central sensitization. These findings not only contribute to understanding the pathogenesis of CIPN but also provide new perspectives for its prevention and treatment. In preclinical models, targeting the pathways of glial activation has demonstrated significant therapeutic effects, suggesting that glial cells represent a promising target for alleviating CIPN.

It is important to note that the activation states of microglia and astrocytes can be broadly categorized into two types: the neurotoxic M1 and A1 phenotypes, and the reparative M2 and A2 phenotypes. These phenotypes are interconvertible and display heterogeneity across different stages of CIPN. Most studies to date have shown that, in the acute phase of CIPN, reactive microglia and astrocytes predominantly adopt pro‐inflammatory phenotypes. Five publications we reviewed provided detailed analyses of glial phenotypic transitions during CIPN progression, demonstrating that with cumulative exposure to chemotherapeutic agents, M1 and A1 phenotypes progressively increase, whereas M2 and A2 phenotypes were scarcely observed.^70^, ^104^, ^107^, ^109^, ^111^ With advances in technologies such as single‐cell RNA sequencing, future studies may enable more refined characterization of these states and their dynamic shifts across cell types and time, thereby offering temporally optimized windows for interventions targeting “phenotypic correction.” By contrast, investigations into glial phenotypic transitions during chronic CIPN remain limited.

Despite recent advances, most of the available evidence is still confined to the preclinical stage. Animal experiments provide essential evidence for identifying therapeutic targets with translational potential, whereas carefully designed preclinical studies enhance the likelihood of successful clinical application. During clinical translation, several key issues warrant close attention. The first is the risk of off‐target effects. Indiscriminate inhibition of glial cells may compromise their critical functions in homeostatic monitoring, synaptic support, and host defense. Consequently, pathway‐selective and stage‐specific regulatory strategies (e.g., S1PR1‐biased modulation or P2X7R antagonism) are favored over broad‐spectrum anti‐inflammatory approaches. The second pertains to the route of administration. Because the DRG lies outside the classical blood–brain barrier and is anatomically more accessible, DRG‐targeted or intrathecal delivery is preferable to maximize target exposure while reducing central off‐target effects. The third involves biomarkers and endpoints. Recent human studies have demonstrated significant elevations in serum neurofilament light chain (NfL) and GFAP following paclitaxel treatment, supporting their use in CIPN risk stratification and as potential pharmacodynamic markers.^182^ In addition, positron emission tomography studies in humans have confirmed translocator protein (TSPO)‐mediated glial activation in chronic pain,^183^ suggesting that once CIPN‐specific TSPO (or non‐TSPO) microglial tracers are validated, they may hold considerable translational potential. Complementary assessment methods include quantitative sensory testing, magnetic resonance neuroimaging/high‐resolution ultrasound, and evaluation of intraepidermal nerve fiber density. It is worth noting that clinical trials investigating minocycline for CIPN prevention have predominantly produced negative or neutral outcomes, despite its robust anti‐glial effects in animal studies.^184^ This underscores the need for mechanism‐based patient stratification, careful consideration of intervention timing (prevention vs. early treatment), and comprehensive endpoint evaluations that integrate symptomatic, functional, and objective biomarker measures.

This review has several limitations. First, most available studies are derived from preclinical experiments in mice or rats, with substantial heterogeneity in dosing regimens, observation periods, and behavioral assays, which complicates cross‐study comparisons. Second, sex differences and the complex interactions occurring during cancer therapy have not been adequately addressed. In terms of human evidence, imaging studies of glial activation in CIPN patients remain scarce. Although advances have been made in biomarker research, validation in larger, multicenter cohorts with standardized protocols and longitudinal follow‐up is still required.

Currently, clinical trials targeting specific glial cell pathways for the treatment of CIPN are underway (Trial registration numbers: ClinicalTrials.gov Identifier: NCI‐2019‐02742; UTN: U1111‐1209‐0075; ANZCTR registration number: ACTRN12618000232235). This review aims to integrate the existing literature and elucidate the mechanisms of glial cells in CIPN, to provide a reference for clinical practice. In summary, we look forward to more effective preventive and therapeutic strategies to alleviate the suffering of patients with CIPN, ensure the smooth implementation of chemotherapy, and improve patients' quality of life.

## AUTHOR CONTRIBUTIONS

Long Gu contributed to the drafting of the manuscript. Yonghuai Feng and Song Cao contributed to the organizational thinking and revision of the paper, finalized the manuscript, and approved the final version for review. All authors read and approved the final version of the manuscript.

## CONFLICT OF INTEREST STATEMENT

Song Cao is a member of the Editorial Board of Ibrain. The author was not involved in the review or decision‐making process for this manuscript.

## ETHICS STATEMENT

Not applicable.

## Data Availability

Not applicable as no new data are generated in this study.
